# Leukocytosis as a prognostic marker in the development of fetal inflammatory response syndrome

**DOI:** 10.3402/ljm.v8i0.21674

**Published:** 2013-10-24

**Authors:** Daiva Bartkeviciene, Ingrida Pilypiene, Grazina Drasutiene, Raminta Bausyte, Mykolas Mauricas, Mindaugas Silkunas, Irena Dumalakiene

**Affiliations:** 1 Vilnius University Clinic of Obstetrics and Gynaecology, Vilnius, Lithuania; 2 Department of Neonatology, Vilnius University Hospital “Santariškių Klinikos,” Vilnius, Lithuania; 3 State Research Institute Center for Innovative Medicine, Vilnius, Lithuania; 4 Department of Chemistry and Bioengineering, Faculty of Fundamental Sciences, Vilnius Gediminas Technical University, Vilnius, Lithuania

**Keywords:** fetal inflammatory response syndrome, intrauterine infection, leukocytes, preterm delivery, IL-6, TNF-α

## Abstract

**Objective:**

To identify and evaluate the correlation between leukocyte count in maternal blood and the risk of developing fetal inflammatory response syndrome (FIRS).

**Patients and methods:**

The study involved 158 infants born at 22−34 weeks of gestation and their mothers. Umbilical cord blood cytokines were evaluated in immunoassay tests and maternal blood was tested for the leukocyte formula.

**Results:**

The period of gestation was significantly shorter in the FIRS group compared to the control group (29.5±3.1 vs. 32.2±2.4 weeks, *p*<0.001). Gestational age was ≤30 weeks for 53.8% of the newborns in the FIRS group and 15.8% of the newborns in the control group (*p*<0.001). The number of leukocytes in maternal blood before and during labor was significantly higher in the FIRS group than in the control group (*p*=0.034 and 0.004, respectively). The study determined the correlation between the total leukocyte count in maternal blood and IL-6 concentration during labor (*p*=0.05) and tumor necrosis factor (TNF-α) concentration in umbilical cord blood before and during labor (*p*=0.02 and 0.007, respectively).

**Conclusion:**

Leukocytosis in the FIRS group was significantly higher than in the control group before and during labor. According to our data, one of the possible indicators of intrauterine infection could be the number of leukocytes in maternal blood.

Preterm delivery is the major cause of perinatal mortality and morbidity in Lithuania, where the rate of preterm deliveries remains approximately 5–6% of all deliveries (1). The rate of mortality is 20–30 times higher in preterm infants than those born at term and accounts for 80% of total neonatal deaths and 40% of infant deaths (1, 2).

The most frequent reason for preterm delivery is intrauterine infection (about 40% of all preterm deliveries). This rate is inversely proportional to gestational age (3–5). Intrauterine infection may cause fetal infection and inflammation that could lead to the heaviest inflammatory response in the fetus, defined by fetal inflammatory response syndrome (FIRS). FIRS is characterized by increase of IL-6 level in fetal blood plasma (≥11 pg/ml) and the presence of umbilical cord inflammation (funisitis) (6, 7). FIRS may cause heavy damage in the fetus and newborns, as well as respiratory distress syndrome, neonatal sepsis, pneumonia, bronchial-pulmonary dysplasia, necrotizing enterocolitis, intraventricular hemorrhage, and periventricular leukomalacia (6). Moreover, fetal exposure to infection may cause fetal death and neonatal sepsis (3, 8–11).

Intrauterine infection is often chronic and non-symptomatic, so it is very important to find markers to help identify pregnant woman at high risk for intrauterine infection, prognosticate the possibility of FIRS development, and prevent the development of threatening complications. Therefore, it is important to determine critical values of inflammatory markers for identification of intrauterine infection that could lead to premature birth.

The aim of this study was to identify and evaluate the correlation between leukocyte concentration in maternal blood and the risk of developing FIRS.

## Patients and methods

This case–control study was performed in Vilnius City Clinical Hospital during 2007–2009. In this third-level hospital, there are about 3,500 births every year (i.e. about 12% of all newborns in Lithuania). About 10–11% of all deliveries are premature.

The study involved 158 infants born at 22–34 weeks of gestation and their mothers. The eligibility criteria were as follows: 1) mother's age >18 years; 2) gestation of 22–34 weeks and 6 days; 3) properly attached placenta; 4) no diabetes, chronic cardiovascular and respiratory system diseases, severe anemia (Hb < 80.00 g/L) or autoimmune or oncological diseases in the mother. The exclusion criterion were the following: 1) stillbirth; 2) alcoholism and/or drug addiction of the woman; 3) malformations in newborn; 4) hemolytic newborn disease; 5) umbilical cord artery blood pH < 7.00; 6) birth trauma in the newborn; 7) mother's refusal to participate in the study.

All newborns and their mothers were investigated according to the same scheme: 1) umbilical cord blood cytokines IL-6 and tumor necrosis factor (TNF-α) were evaluated in each newborn; 2) depending on the concentration of umbilical cord blood's IL-6, the newborns were assigned to FIRS (IL-6 ≥ 11 pg/ml) or control (IL-6 < 11 pg/ml) group; 3) each mother was tested for total leukocyte count and leukocyte formula of the blood before, during, and after labor; 4) the correlation between the number of leukocytes in maternal blood and concentration of various cytokines in umbilical cord blood was evaluated; 5) the correlation between leukocytosis in maternal blood before, during, and after labor and the development of FIRS was evaluated. In addition, the culture of cervical secretions, C-reactive protein (CRP) in maternal blood, histological placenta study, and bacteriologic newborn blood test were performed.

Immunological studies of cord blood cytokines were performed at the State Research Institute Center for Innovative Medicine. After birth, the umbilical blood cord vein was punctured and 5 ml of blood was taken into a vacuum tube. Within 1 h, the blood sample was centrifuged for 14 min at 1,500 rpm/min and plasma was immediately frozen at –80°C until assayed. IL-6 and TNF-α levels in umbilical cord blood plasma were measured with commercially available enzyme-linked immunosorbent assay (ELISA) kits (Bender MedSystems, Austria) according to the manufacturer's protocols. A special program calculated the amount of cytokines for ELISA (Gen5 Microplate Data Collection & Analysis Software; BioTek Instruments, USA).

Statistics were performed using the SPSS statistics software version 15.0. Data are presented as mean±SD, median, minimum, and maximum. Quantitative variables of two independent groups were compared by the parametric Student *t*-test and non-parametric Mann–Whitney test. For assessing the correlation between results, the Spearman test was used. *p*<0.05 were considered significant. The specificity and sensitivity of testing were assessed by analyzing receiver operator characteristic curves (ROC).

The study was performed with permission No. 14 approved by the Lithuanian Bioethics Committee as of 20 June 2007.

## Results

### The participants

The average age of the mothers in the FIRS group was 27.3±6.5 years and in the control group 28.3±5.2 years, but the difference was not significant (*p*>0.05). The level of CRP in maternal blood before, during, and after labor was significantly higher in the FIRS group compared to the control group (*p*<0.001). The study determined the correlation between CRP in maternal blood and IL-6 concentration during all prenatal periods (*p*<0.001). The culture of the cervical secretions was done during the perinatal period for 42% of the mothers. The occurrence of a positive culture in the FIRS group was not significantly different from the control group (*p*>0.05). In the histological placenta study, inflammatory changes in placenta were detected in 40.3% of the cases studied. The frequency of histological chorioamnionitis without funisitis and deciduitis did not differ between the two groups (*p*>0.05). Histological chorioamnionitis with funisitis was found in 51% of placentas in the FIRS group, while the control group demonstrated such changes in only 1% of placentas (*p*<0.001). No pathological changes were detected in 10% of placentas in the FIRS group.

The sex of newborns was distributed almost equally between boys and girls: 49.4% of newborns were boys and 50.6% were girls (*p =*0.82). The average period of gestation was 31.3±2.9 weeks, and it was significantly (*p*<0.001) shorter in the FIRS group (29.5±3.1 weeks) than in the control group (32.2±2.4 weeks). Gestational age was ≤ 30 weeks in 53.8% of the newborns in the FIRS group and 15.8% of the newborns in the control group (*p*<0.001). The mean weight of the FIRS group newborns was 1,424.7±564.0 g and of the control group 2,006.3±554.1 g (*p*<0.001). The average umbilical cord artery blood pH in newborns was 7.3±0.1; no difference between groups was observed (*p* >0.05). Bacteriologic blood test was performed on 139 newborns. Positive blood cultures were found in one newborn from the FIRS group and one from the control group (*p*=1.0). *Lysteria monocytogenes* was cultured in the blood sample of the newborn from the FIRS group, and *Klebsiella pneumonia* from the blood sample of one from the control group.

### The total leukocyte count and leukocyte formula in maternal blood

In this study we compared the leukocyte count in maternal blood sample before, during, and after labor (Table 1).

**Table 1:** Comparison of the number of leukocytes in maternal blood

The number of leukocytes	FIRS group *n =* 52	Control group *n =* 106	*p*†
before labor (×10^9^/L)			
Mean±SD	**13.20±4.80**	11.24±3.14	
Median	**12.74**	11.43	**0.034**
[min – max]	[6.53–25.52]	[3.62–25.46]	
during labor (×10^9^/L)			
Mean±SD	**14.04±4.80**	11.3±3.74	
Median	**13.31**	11.61	**0.004**
[min – max]	[6.53–25.34]	[5.73–26.30]	
after labor (×10^9^/L)			
Mean±SD	12.93±4.64	12.12±3.73	
Median	12.23	11.65	0.565
[min – max]	[6.73–24.88]	[5.73–25.46]	

† Mann–Whitney test; FIRS, fetal inflammatory response syndrome; SD, standard deviation.

Significant values are set in bold.

**Fig. 1:**
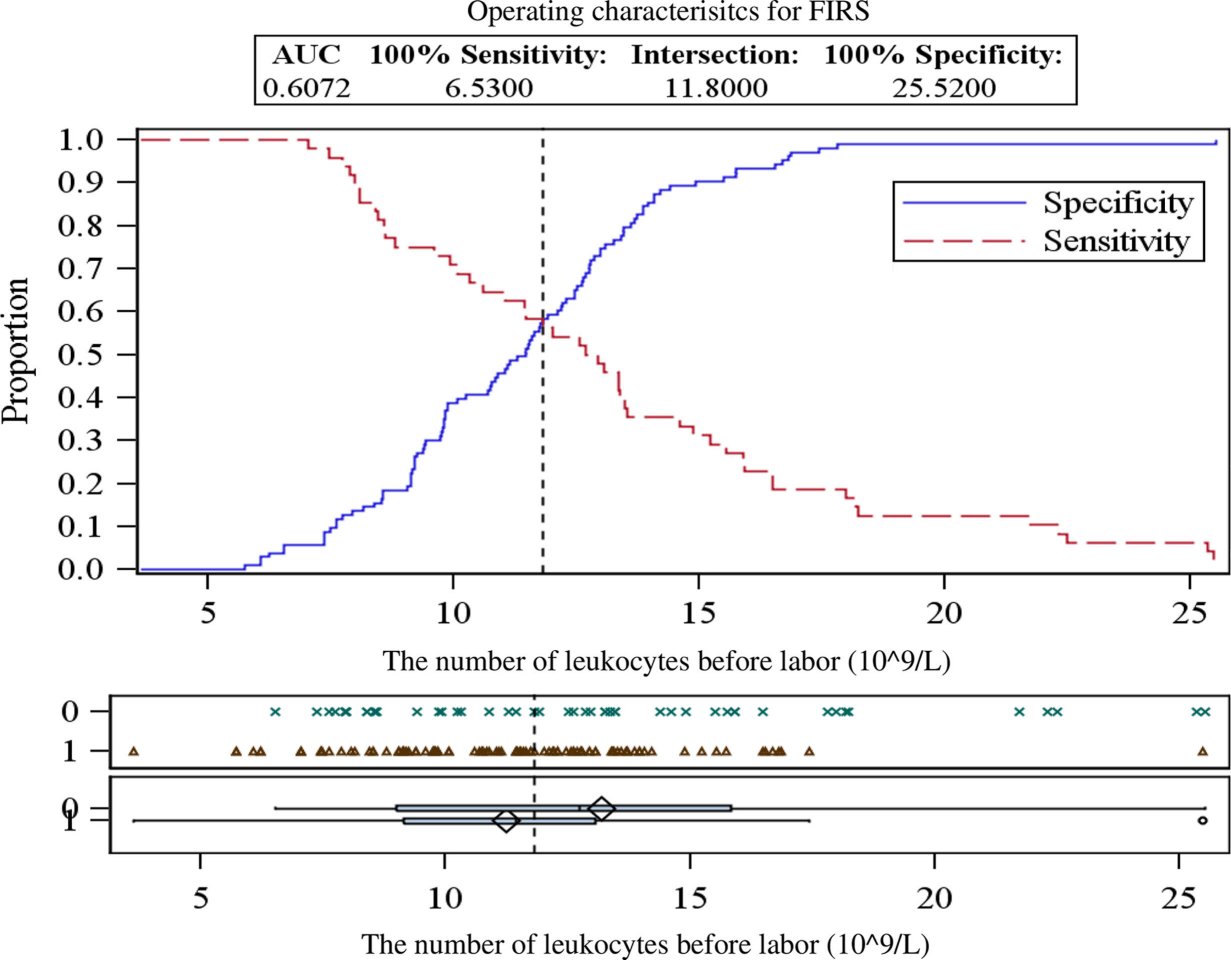
ROC curves analysis for determining the critical values of leukocytes in maternal blood before labor.

**Fig. 2:**
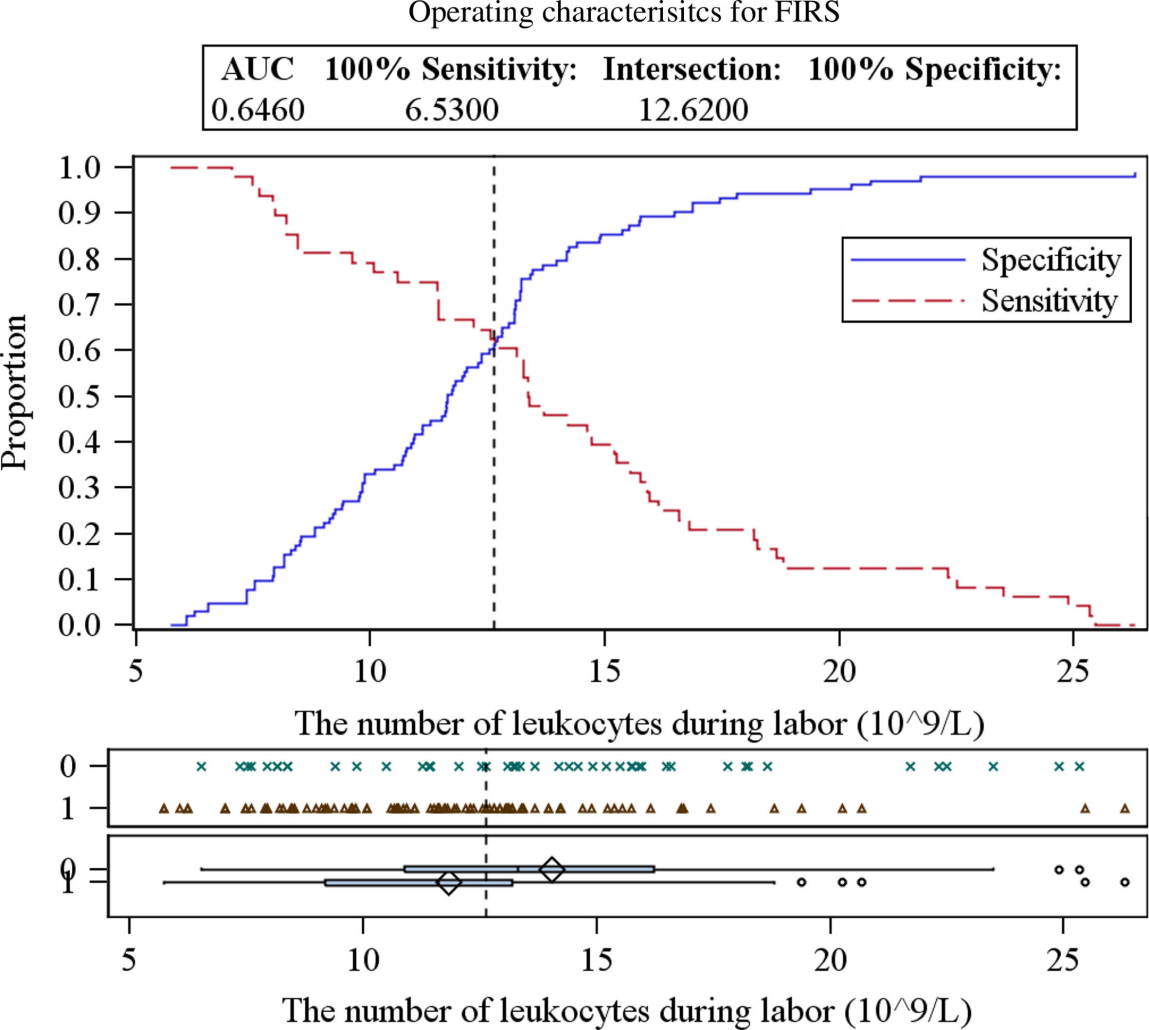
ROC curves analysis for determining the critical values of leukocytes in maternal blood during labor.

While examining maternal blood samples during different periods, elevated levels of leukocytes before (13.20±4.80×10^9^/L) and during labor (14.04±4.80×10^9^/L) were detected in the FIRS group (Table 1). Total leukocyte count of these FIRS stages was significantly higher than in the control group (*p*=0.034 and 0.004, respectively). The critical values of leukocytes in maternal blood for predicting the development of FIRS are presented in Figs. 1 and 2 and in Table 2. No significant difference was detected between other blood cell fractions – erythrocytes and platelets – between the groups (*p* >0.05).

**Table 2:** The critical values of leukocytes in maternal blood for predicting the development of fetal inflammatory response syndrome

The number of leukocytes	Critical value (×10^9^/L)	Sensitivity (%)	Specificity (%)	95% CI	AUC	PPV (%)	NPV (%)
Before labor	11.81	56.3	58.3	0.5030–0.7118	0.6074	74.1	38.6
During labor	12.64	60.4	62.1	0.5454–0.7467	0.6460	77.1	42.6
After labor	12.05	52.1	53.4	0.4245–0.6338	0.5291	70.5	34.2

CI, confidence Interval; AUC, area under the ROC curve; PPV, positive predictive value; NPV, negative predictive value.

### The concentration of cytokines in umbilical cord blood

Each newborn was tested for umbilical cord blood cytokines IL-6 and TNF-α. The study determined correlation between the total leukocyte count in maternal blood and IL-6 concentration in umbilical cord blood during labor (*p*=0.05) (Table 3).

**Table 3:** Correlation between the number of leukocytes in maternal blood and concentrations of cytokines in umbilical cord blood

	Spearman correlation coefficient, r (*p* value)	
	
The number of leukocytes	IL-6	TNF-α
before labor	0.1 (0.1)	**0.2 (0.02)**
during labor	**0.2 (0.05)**	**0.2 (0.007)**
after labor	−0.04 (0.6)	0.1 (0.1)

Significant values are set in bold.

Also, a significant difference was detected between the leukocytes count in maternal blood and TNF-α concentration in umbilical cord blood before and during labor (*p*=0.02 and 0.007, respectively) (Table 3).

## Discussion

Leukocytosis is characterized by an elevated number of white cells in the blood (>11.00×10^9^/L). Leukocytosis may be physiological, caused by fasting, pregnancy, strong emotions or high stress, or pathological due to the bone marrow's response to inflammation or infection.

Pregnancy causes a great variety of changes in pregnant woman organism and is mostly associated with suppression of humoral and cellular immune functions in order to tolerate the ‘foreign’ semiallogeneic fetal graft (12). One of the most important mechanisms, which continue throughout pregnancy, involves the suppression of IL-2, IFN-γ, TNF-β, polymorphonuclear leukocyte chemotaxis, and adherence functions (12).

On the contrary, not all aspects of immunological function are depressed. For example, there is up-regulation of Th2 cells to increase the secretion of IL-4, IL-6, and IL-13 (12). In cervical mucus, peak levels of immunoglobulins A and G are significantly higher in pregnant women compared with normally menstruating women (13). The amount of IL-1β found in cervical mucus during pregnancy is approximately 10-fold greater than in the non-pregnant state (12, 14).

According to different studies, the leukocyte count during normal pregnancy varies from 5.00 to 12.00×10^9^/L (12). Moreover, during labor and the early postnatal period it may become considerably elevated, up to 25.00×10^9^/L or even more (averages 14.00–16.00 ×10^9^/L) (12). It is noted that the percentages of granulocytes and CD8 T lymphocytes are significantly increased during the third trimester. At the same time, the percentages of CD4 T lymphocytes and monocytes decrease (12). So it is often said that the leukocyte count in maternal blood during the perinatal period is not a reliable marker for diagnosing intrauterine infection as the cause of premature delivery (1).

In this study, we found that the average of the leukocyte count in maternal blood was higher in both groups than the normal range (4.50–10.00×10^9^/L). In the FIRS group, leukocytosis was significantly higher before and during labor than in the control group (*p*=0.034 and 0.004, respectively). Also, we identified a correlation (*p*=0.05) between leukocytes count in maternal blood during labor and IL-6 in umbilical cord blood. Moreover, we determined the association between leukocytosis in mothers’ blood before and during labor and TNF-α in umbilical cord blood (*p*=0.02 and 0.007, respectively).

IL-6 and TNF-α are very important in premature delivery. IL-6 is an acute inflammatory response phase marker that induces the secretion of various acute phase proteins (e.g. CRP), and increases in the mother's blood and amniotic fluid, if premature delivery is caused by intrauterine infection (2, 15, 16). Moreover, elevation of IL-6 levels is part of the definition of FIRS (6, 7). Another important factor is TNF-α, which stimulates prostaglandin synthesis in the chorioamniotic membranes, decidual plate and uterus muscles. In the presence of TNF-α, metalloproteases and other bioactive substances are released. The prostaglandins stimulate uterine contractions while the metalloproteases have a strong effect on the chorioamniotic membranes, leading to their rupture (2, 17–21).

Although the cultures from the cervix were taken from only 42% of the mothers, positive results were found in up to 40% of the cultures. The number of positive cultures in the FIRS and the control groups were similar. This is because microbial invasion of amniotic fluid is often chronic and clinically silent in the mid-trimester of pregnancy, culminating in premature labor (22, 23). Different studies point out that an increased level of cytokines in a woman's blood and histological chorioamnionitis are associated with the woman's inflammatory reaction, while an increased concentration of inflammatory cytokines in fetal blood and funisitis show that the fetus is involved in this reaction, i.e. processes described as FIRS take place (7, 9, 24). According to our data, histological inflammatory changes in the placenta were detected in 40% of the cases studied (25). Similar results have been demonstrated by others as well (24). The frequency of histological chorioamnionitis and deciduitis, which would confirm an inflammatory reaction initiated by the mother's body, did not differ between the groups. Histological chorioamnionitis with funisitis was found in 51% of placentas in the FIRS group but in only 1% of placentas in the control group, which confirms close associations between FIRS and funisitis.

In our study, bacteriologically confirmed neonatal sepsis was diagnosed in two newborns (1.3%). One was in the FIRS group and the other in the control group, so no correlation was found between FIRS and early neonate sepsis. According to the published scientific data, the frequency of early neonate sepsis in newborns younger than 30 weeks of gestation accounts for about 7% (26). Alabama preterm delivery data suggest that *Ureaplasma urealyticum* and *Mycoplasma hominis* were detected in umbilical cord blood of 23% of very early preterm neonates (27), but in Lithuania these intrauterine infectious agents are rarely detected in newborns’ blood.

## Conclusion

Leukocytosis in the FIRS group was significantly higher than in the control group before and during labor. According to our data, one of the possible indicators of intrauterine infection could be the number of leukocytes in maternal blood.
